# Prevalence of Carnitine Deficiency and Decreased Carnitine Levels in Patients on Peritoneal Dialysis

**DOI:** 10.3390/nu11112645

**Published:** 2019-11-04

**Authors:** Satoshi Shimizu, Hiroyuki Takashima, Ritsukou Tei, Tetsuya Furukawa, Makiyo Okamura, Maki Kitai, Chinami Nagura, Takashi Maruyama, Terumi Higuchi, Masanori Abe

**Affiliations:** 1 Division of Nephrology, Hypertension and Endocrinology, Department of Internal Medicine, Nihon University School of Medicine, 30-1 Oyaguchi Kami-cho, Itabashi-ku, Tokyo 173-8610, Japan; zeejyo@gmail.com (S.S.); craftsmanship.t.h@gmail.com (H.T.); haru_li_huang@yahoo.co.jp (R.T.); tetsuf0605@gmail.com (T.F.); makiyo91supika@yahoo.co.jp (M.O.); kitai.maki@nihon-u.ac.jp (M.K.); nagura.chinami@nihon-u.ac.jp (C.N.); maruyama.takashi@nihon-u.ac.jp (T.M.); 2 Department of Nephrology, Keiai Hospital, 3-10-6, Mukaihara, Itabashi-ku, Tokyo 173-0036, Japan; thiguchi@keiai-hospital.jp

**Keywords:** carnitine, peritoneal dialysis, hemodialysis

## Abstract

Background: Carnitine deficiency is common in patients on dialysis. Serum free carnitine concentration is significantly lower in patients on hemodialysis (HD) than in healthy individuals. However, there are few reports on serum free carnitine concentration in patients on peritoneal dialysis (PD). Methods: We examined serum concentrations of total, free, and acylcarnitine and the acylcarnitine/free carnitine ratio in 34 PD and 34 age-, sex-, and dialysis duration-matched HD patients. We investigated the prevalence of carnitine deficiency and clinical factors associated with carnitine deficiency in the PD group. Results: Prevalence of carnitine deficiency was 8.8% in the PD group and 17.7% in the HD group (*p* = 0.283). High risk of carnitine deficiency was found in 73.5% of the PD group and 76.4% of the HD group (*p* = 0.604). Carnitine insufficiency was found in 82.3% of the PD group and 88.2% of HD group (*p* = 0.733). Multivariate analysis revealed that duration of dialysis and age were independent predictors of serum free carnitine level in the PD group. Conclusions: The prevalence of carnitine deficiency, high risk of carnitine deficiency, and carnitine insufficiency in PD patients was 8.8%, 73.5%, and 82.3%, respectively. These rates were comparable to those in patients on HD.

## 1. Introduction

Carnitine is a naturally-occurring substance necessary for transporting long-chain fatty acids into the mitochondria and plays an important role in energy supply in the body. Its other major actions in vivo include suppression of apoptosis, correction of cytotoxicity by excessive acyl groups, and stabilization of the erythrocyte membrane [1,2,3,4,5,6]. Carnitine is synthesized from the two amino acids lysine and methionine in the liver and kidney.

Carnitine deficiency has been reported to be highly prevalent in patients on hemodialysis (HD) [7,8]. The causes of carnitine deficiency in these patients include insufficient carnitine intake, decreased biosynthesis, removal by HD, and loss of preferential renal excretion of acylcarnitine. However, there have been few such reports in patients on peritoneal dialysis (PD), though normal plasma and muscle carnitine concentrations have been reported in this population [9]. A decrease in plasma free carnitine and an increase in acylcarnitine/free carnitine ratio has also been reported in patients on PD compared with age- and sex-matched controls [10,11]. Several mechanisms may be involved in the development of carnitine deficiency in patients on PD. First, decreased renal carnitine synthesis has been shown to influence carnitine deficiency, in addition to decreased renal excretion of acylcarnitine [12,13]. Second, loss of free carnitine into PD fluid may also be involved [14]. Third, patients on PD require a diet with restricted intake of foods containing carnitine because of kidney dysfunction [12,13].

Carnitine deficiency is associated with various pathological conditions, including anemia, cardiac dysfunction, and muscle weakness [15]. Therefore, it is important to promptly detect carnitine deficiency and ensure appropriate carnitine supplementation in patients on dialysis. However, there are few reports of serum carnitine concentration in patients on PD and clinical data on carnitine deficiency is currently limited. Therefore, the prevalence of carnitine deficiency in patients on PD needs to be investigated. In this study, we aimed to clarify the prevalence and clinical characteristics of carnitine deficiency in patients on PD compared with HD.

## 2. Materials and Methods

### 2.1. Patients

The study population comprised patients who were on PD in May 2019. The inclusion criteria were age ≥18 years, duration of PD >6 months at enrollment, and patients for whom medical decisions were made at the participating hospitals. The exclusion criteria were concurrent infectious disease, hepatic disease (chronic liver injury, such as hepatitis and cirrhosis), or malignancy; steroid or immunosuppressant therapy; current hospitalization; and levocarnitine treatment within the past 6 months. After these exclusions, 34 patients were included in the study (PD group). We then compared these patients with 34 age-, sex-, and dialysis duration-matched HD patients (HD group) in our hospital. All patients in the HD group underwent HD sessions (4–5 h each) 3 times weekly. The study protocol was approved by the ethics committee of Keiai Hospital and all procedures fully adhered to the Declaration of Helsinki. The study was registered with the University Hospital Medical Information Network (UMIN000025327). All participants provided written informed consent.

### 2.2. Data Collection

Demographic data and medical histories were collected, including age, sex, dialysis vintage, height, body weight, history of cardiovascular disease, and laboratory parameters. Cardiovascular disease was defined as a history of severe cardiac failure, myocardial infarction, angina pectoris, peripheral artery disease, or stroke. Measurements were made following routine clinical chemistry procedures with commercial kits, including blood cell counts and levels of serum urea nitrogen, serum creatinine, total protein, albumin, electrolytes, total cholesterol, high-density lipoprotein cholesterol, triglycerides, serum iron, total iron binding capacity, serum ferritin, and serum zinc. Latex agglutination immunoassay was used to measure C-reactive protein and serum β_2_-microglobulin. All patients were treated with an erythropoiesis-stimulating agent (ESA; darbepoetin alfa). Monthly ESA dose was recorded. Total weekly Kt/V urea, peritoneal weekly Kt/V urea, renal weekly Kt/V urea in the PD group, and single-pool Kt/V for urea in the HD group were also measured. Enzyme cycling assay was used to measure total and free carnitine concentrations [16]. Acylcarnitine was calculated as total carnitine—free carnitine, and the acyl/free carnitine ratio was then calculated. Normal range was defined as 45 μmol/L to 91 μmol/L for total carnitine, 36 μmol/L to 74 μmol/L for free carnitine, 6 μmol/L to 23 μmol/L for acyl carnitine, and <0.4 for acyl/free carnitine ratio. The prevalence of carnitine deficiency was then investigated. A free carnitine level <20 μmol/L was defined as carnitine deficiency, a level in the range of 20–36 μmol/L as high risk of carnitine deficiency, and an acylcarnitine/free serum carnitine ratio >0.4 as carnitine insufficiency, according to Japanese guidelines. These guidelines were jointly prepared by the Japan Pediatric Society, Japanese Society for Dialysis Therapy, Japan Society of Hepatology, and Japanese Society of Severe Motor and Intellectual Disabilities [17]. A high risk of carnitine deficiency was defined as an extremely high possibility of developing carnitine deficiency [17]. Blood samples were obtained before the start of the first HD session each week in the HD group.

### 2.3. Statistical Analysis

Data are shown as mean ± SD or median (interquartile range) as appropriate. Continuous variables were compared using the Student t test or Mann–Whitney U test. Categorical variables were compared using the chi-square test or Fisher’s exact test as appropriate for the data distribution. Factors associated with serum free carnitine level were assessed using multivariable linear regression. Sex, age, duration of PD, serum urea nitrogen, creatinine, hemoglobin, albumin, calcium, phosphate, C-reactive protein, high-density lipoprotein-cholesterol, β2-microglobulin, and total Kt/V were selected for the multivariable model. Statistical significance was set at *p* < 0.05. All analyses were performed using JMP version 12 software (SAS Institute Ltd., Cary, NC, USA).

## 3. Results

Background characteristics of patients in the PD and HD groups are summarized in Table 1. There was no significant difference in age, sex, dialysis duration, causes of end-stage kidney disease, and medications between the two groups. Anuria was present in eight patients in the PD group and in 15 patients in the HD group.

Table 2 compares the laboratory findings between the two groups. There were significant differences in total protein, serum albumin, sodium, potassium, total cholesterol, and HDL cholesterol levels between the PD and HD groups. Conversely, there were no significant differences in other laboratory findings such as hemoglobin, transferrin saturation, zinc, and β_2_-microglobulin levels.

**Figure 1:**
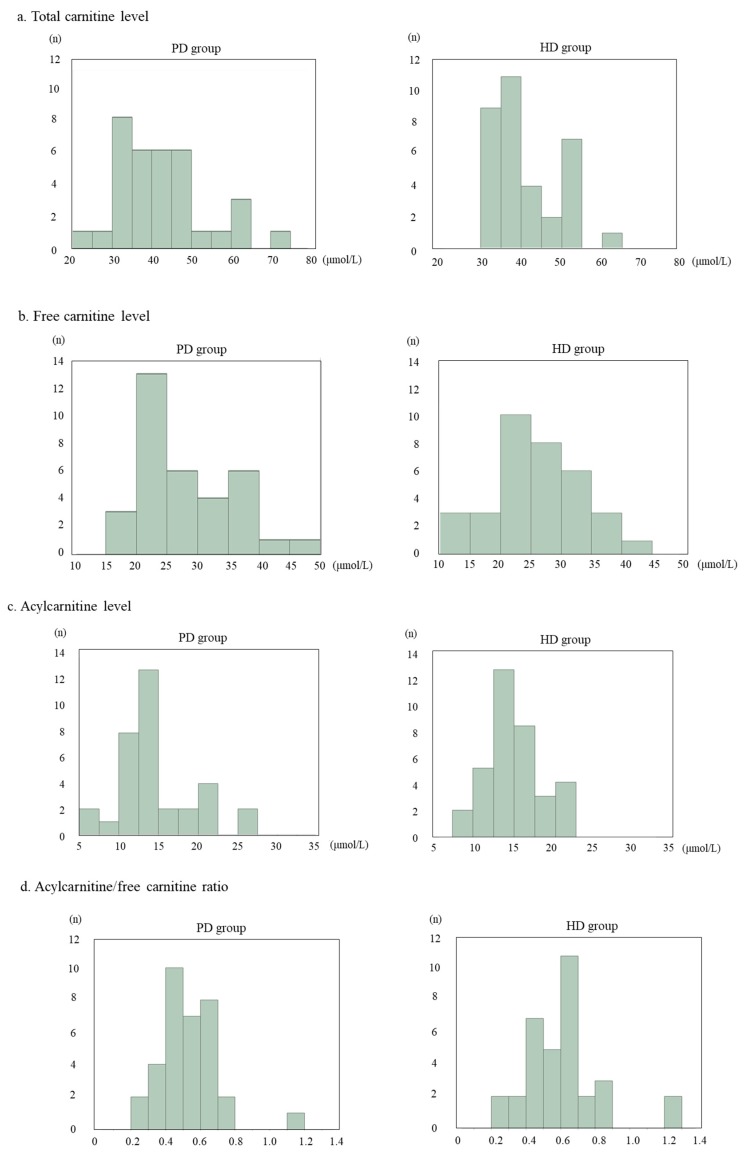
Histogram of carnitine concentrations in the peritoneal dialysis (PD) and hemodialysis (HD) groups. (**a**) Serum total carnitine concentrations in 34 patients on dialysis. (**b**) Serum free carnitine concentrations in the PD and HD groups. (**c**) Serum acylcarnitine concentrations in the PD and HD groups. (**d**) Acylcarnitine/free carnitine ratio in the PD and HD groups. HD, hemodialysis; PD, peritoneal dialysis.

Carnitine concentrations were compared between the PD and HD groups. The distribution of serum total carnitine concentrations was not significantly different between the two groups (PD vs. HD: 42.5 ± 11.2 μmol/L vs. 41.0 ± 8.2 μmol/L; *p =* 0.535; Figure 1a). In addition, the distribution of serum free carnitine concentrations was not significantly different between the groups (PD vs. HD: 28.0 ± 7.8 μmol/L vs. 25.6 ± 7.4 μmol/L; *p* = 0.203; Figure 1b). No significant difference between the groups was noted in the distribution of serum acylcarnitine concentrations (PD vs. HD: 14.5 ± 4.8 μmol/L vs. 15.0 ± 3.3 μmol/L; *p =* 0.634; Figure 1c) or the distribution of serum acylcarnitine/free carnitine ratios (PD vs. HD: 0.52 ± 0.16 vs. 0.62 ± 0.22; *p* = 0.069; Figure 1d).

**Figure 2:**
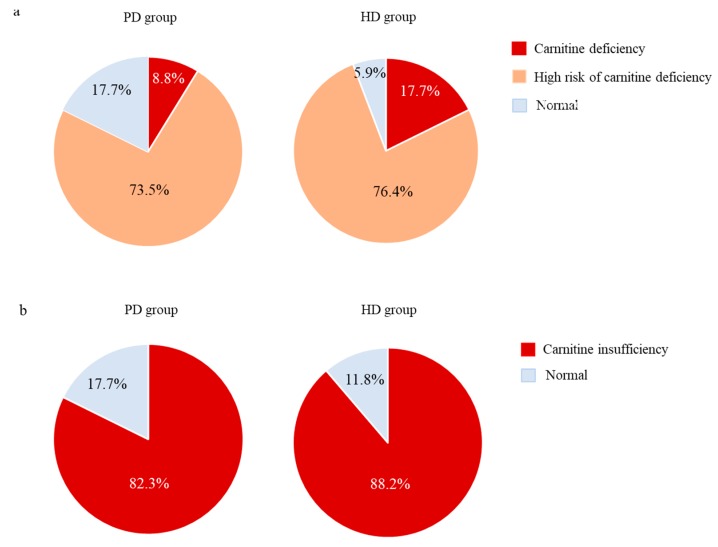
(**a**) Distributions of carnitine deficiency and high risk of carnitine deficiency in the two groups. (**b**) Rate of carnitine insufficiency in the two groups.

Figure 2a compares prevalence of carnitine deficiency and high risk of carnitine deficiency between the two groups. The prevalence of carnitine deficiency was 8.8% in the PD group and 17.7% in the HD group (*p* = 0.283). High risk of carnitine deficiency was found in 73.5% of the PD group and 76.4% in the HD group (*p* = 0.604). Although 17.7% of PD patients had normal carnitine concentrations, only 2 patients (5.9%) in the HD group did (*p* = 0.283). Figure 2b shows the acylcarnitine/free carnitine ratio in the two groups. Carnitine insufficiency was 82.3% in the PD group and 88.2% in the HD group (*p* = 0.733). The prevalence rate of carnitine deficiency, high risk of carnitine deficiency, and carnitine insufficiency were comparable in both groups.

Multivariate analysis revealed that the duration of dialysis and age were independent predictors of serum free carnitine level in the PD group (Table 3).

## 4. Discussion

In this observational study, we found that the prevalence of carnitine deficiency was 8.8% and the rate of carnitine insufficiency was 82.3% in patients on PD. Furthermore, these rates were not significantly different from those of the HD group. We found that carnitine deficiency was correlated with age and duration of PD. To our knowledge, this is the first report to show the prevalence and clinical characteristics of carnitine deficiency in patients on PD compared with matched HD patients.

A decrease in plasma free carnitine concentrations and an increase in acylcarnitine/free carnitine ratio was reported in patients on PD compared with matched healthy controls [18]. Deficiency of plasma free carnitine, however, has been found to be generally less marked than in patients on HD [19]. It has also been reported that carnitine deficiency is likely to occur after long-term dialysis in patients on HD [20,21]. We found that age and duration of dialysis were related to carnitine deficiency in PD patients as well. Carnitine deficiency was more severe in those with lower blood urea nitrogen levels and those on PD, but not in patients on HD [22]. The molecular weight of carnitine is 161 Da and it is not bound to protein in the blood. Therefore, plasma carnitine levels might be associated with serum urea nitrogen levels because the carnitine removal rate is considered to be similar to that of blood urea nitrogen [8]. Furthermore, a lower blood urea nitrogen level suggests deficient protein intake. In our study, no correlation was found between serum albumin levels and carnitine concentrations and this was consistent with previous studies [22]. These previous studies included relatively younger patients such as children on PD [10,11,18,19]. However, our study included older patients on PD. Therefore, our findings showed comparable results in terms of total, free, and acylcarnitine concentrations in both the PD and HD groups because intake of foods containing carnitine and muscle volume in our PD population were different from those of previous studies.

According to Leschke et al., the carnitine removal rate in HD is lower than the continuous ambulatory peritoneal dialysis (CAPD) elimination rate and the carnitine excretion efficiency of CAPD is nearly twice that of hemodialysis [23]. In contrast, loss of carnitine has been reported to be greater in patients on HD than in patients on PD (1078 ± 14.3 μmol/week in CAPD patients and 1518 ± 273 μmol/week in HD patients) [10]. Patients on HD showed rapid and relatively large decreases in plasma concentrations of carnitine, which returned to the normal range within about 6 h after HD treatment. Muscle carnitine concentrations were shown to be significantly lower in HD patients than in nondialysis patients, thus supporting the notion that HD depletes not only plasma, but also muscle stores of carnitine. Patients on CAPD, a continuous and gradual process, showed weekly losses of carnitine that were lower than those in patients on HD. We previously reported that a single HD session resulted in a 64 ± 4% reduction rate of serum free carnitine [8]. Furthermore, the PD technique used might influence carnitine metabolism in patients with end-stage kidney disease. Lower levels of free and acyl carnitine in plasma have been reported in patients on automated PD compared with in patients on CAPD. Automated PD is characterized by shorter and larger dwell volumes than CAPD, which might favor the removal of carnitine [24]. In the present study, however, we could not measure carnitine concentrations in the PD fluid, so further studies are needed to clarify these discrepancies.

Carnitine deficiency occurred frequently in patients on PD as well as those on HD. The clinical features include severe and persistent muscle cramps or hypotension during dialysis, low energy affecting quality of life, skeletal muscle weakness or myopathy, cardiomyopathy, and anemia of uremia unresponsive to or requiring large doses of ESA. Levocarnitine supplementation might improve cardiac function, brachial-ankle pulse wave velocity, ESA-resistant anemia, and maintenance of physical function in dialysis patients [25,26,27,28,29]. Levocarnitine supplementation may also cause symptomatic improvement due to carnitine deficiency in patients on PD as well as in patients on HD. However, there are limited clinical data on the effects of levocarnitine therapy in patients on PD. The results of a 3 month course of levocarnitine therapy on several indices have been shown to be related to renal anemia [11]. One study showed improved erythropoietin-resistant anemia following carnitine supplementation in 12 patients on PD. However, another study reported no improvement in erythropoietin-resistant anemia by carnitine supplementation in 12 infants who were on PD [30]. Therefore, larger interventional studies are required to investigate the effects of carnitine supplementation for erythropoietin-resistant anemia in patients on PD.

Glucose is the standard osmotic agent used in dialysate for PD. It has a molecular weight of 180 Da, high osmotic capacity, low cost, and an acceptable safety profile. It also represents an energy source for patients who are often not well nourished. However, prolonged exposure to high glucose content in PD solution is associated with accelerated progressive changes in peritoneal membrane function, which causes ultrafiltration failure [31]. Furthermore, excessive intraperitoneal glucose absorption has the potential to cause many systemic metabolic side effects in PD patients, including insulin resistance, new onset diabetes, and cardiovascular disease [32,33,34]. Therefore, several alternative osmotic agents have been examined over the years, but only two agents are currently available for use in glucose-free solutions for PD in clinical practice—icodextrin and amino acids. However, both icodextrin and amino acids can only replace 30%–50% of the daily glucose absorption, and their use is limited to a single daily peritoneal exchange [35,36]. Recently, carnitine-enriched PD solutions were used to demonstrate the effectiveness of L-carnitine as an efficient osmolyte in PD and its favorable effect on insulin sensitivity in patients [37]. L-carnitine as an osmotic-metabolic agent in PD solution represents a novel tool for combating glucose-associated toxicity.

Our study has several limitations. First, the number of patients on PD was relatively small. Further studies are thus needed to compare the prevalence of carnitine deficiency between PD and HD populations. Second, we could not measure the removal amounts of carnitine by PD using the dialysate, and so there is need for more comparisons of serum carnitine loss in patients on PD and HD. Third, the study design was cross-sectional and observational, so we cannot conclusively state the importance of carnitine supplementation. Because the number of older patients on dialysis is increasing, the incidence of carnitine deficiency may also increase. It is thus necessary to conduct further investigations on the efficacy of carnitine treatment for patients on PD with carnitine deficiency.

## 5. Conclusions

In conclusion, the prevalence of carnitine deficiency, high risk of carnitine deficiency, and carnitine insufficiency in PD patients was 8.8%, 73.5%, and 82.3%, respectively. These rates were comparable to those in patients on HD.

## Figures and Tables

**Table 1:** Characteristics and medications at baseline in the PD and HD groups.

Variables	PD Group	HD Group	*p* Value
*N* (Male/Female)	34 (25/9)	34 (25/9)	1.000
Age (years)	61.5 ± 16.4	64.2 ± 12.8	0.467
Duration of dialysis(m)	13.0 (8.5–29.5)	19.0 (8.0–54.0)	0.122
History of CVD *n* (%)	5 (14.7)	6 (17.6)	0.742
Smoking, *n* (%)	6 (17.7)	7 (20.6)	0.758
Alcohol use, *n* (%)	5 (14.7)	7 (20.6)	0.524
Systolic BP (mmHg)	141 ± 15	143 ± 14	0.661
Diastolic BP (mmHg)	81 ± 10	80 ± 10	0.752
Heart rate (bpm)	72 ± 10	73 ± 10	0.706
Body mass index (kg/m^2^)	23.1 ± 4.0	22.2 ± 4.0	0.402
Anuria, *n* (%)	9 (26.5)	15 (44.1)	0.128
Causes of ESKD, *n* (%)			0.694
Diabetes mellitus	10 (29.5)	8 (23.5)	
Chronic glomerular nephritis	8 (23.5)	9 (26.5)	
Nephrosclerosis	13 (38.2)	11 (32.3)	
Others	3 (8.8)	6 (17.7)	
Medications, *n* (%)			
RAS inhibitor	25 (73.5)	28 (82.3)	0.380
Active vitamin D	22 (64.7)	25 (73.5)	0.431
Phosphate binders	29 (85.3)	31 (91.2)	0.452
Statin	12 (35.2)	15 (44.1)	0.461

BP, blood pressure; CVD, cardiovascular disease; ESKD, end-stage kidney disease; HD, hemodialysis; PD, peritoneal dialysis; RAS, renin-angiotensin system.

**Table 2:** Clinical and laboratory parameters in the PD and HD groups.

Variables	PD Group	HD Group	*p* Value
sUN (mg/dL)	51 ± 15	56 ± 13	0.180
Creatinine (mg/dL)	8.9 ± 3.8	10.1 ± 3.3	0.214
Total protein (g/dL)	6.3 ± 0.7	6.6 ± 0.5	0.038 *
Albumin (g/dL)	3.3 ± 0.5	3.5 ± 0.5	0.018 *
Sodium (mEq/L)	138 ± 4.0	141 ± 3.1	0.001 **
Potassium (mEq/L)	4.3 ± 0.6	4.8 ± 0.7	0.002 **
Calcium (mg/dL)	9.0 ± 0.6	9.0 ± 0.8	0.788
Phosphate (mg/dL)	5.6 ± 1.4	5.2 ± 1.1	0.211
CRP (md/dL)	0.11 (0.1–0.6)	0.11 (0.1–0.5)	0.976
Total-cholesterol (mg/dL)	180 ± 40	153 ± 32	0.004 **
HDL-cholesterol (mg/dL)	55 ± 16	36 ± 15	<0.001
Triglyceride (mg/dL)	94 (70–140)	115 (88–163)	0.300
Hemoglobin (g/dL)	10.9 ± 1.3	10.8 ± 0.9	0.735
Iron (μg/dL)	83 ± 32	84 ± 33	0.906
TSAT (%)	33.1 ± 16.5	35.4 ± 14.7	0.752
Ferritin (ng/mL)	114 (37–185)	94 (50–151)	0.622
Zinc (μg/dL)	58 ± 10	56 ± 8	0.413
ESA (μg/m)	120 (50–172)	120 (80–160)	0.065
β_2_-MG (mg/L)	25 ± 11	26 ± 8	0.816
Kt/V renal	0.15 (0.05–0.96)	-	-
Kt/V PD	1.1 ± 0.4	-	-
Kt/V total	1.7 ± 0.7	-	-
Kt/V HD	-	1.3 ± 0.2	-
Total carnitine (μmol/L)	42.5 ± 11.2	41.0 ± 8.2	0.535
Free carnitine (μmol/L)	28.0 ± 7.8	25.6 ± 7.4	0.203
Acyl carnitine (μmol/L)	14.5 ± 4.8	15.0 ± 3.3	0.634
Acyl/free carnitine ratio	0.52 ± 0.16	0.62 ± 0.22	0.069

β_2_-MG, β_2_-microglobulin; CRP, C-reactive protein; ESA, erythropoiesis stimulating agent; HD, hemodialysis; HDL, high-density lipoprotein; PD, peritoneal dialysis; sUN, serum urea nitrogen; TSAT, transferrin saturation. * *p* < 0.05, ** *p* < 0.01.

**Table 3:** Results of multiple regression analysis of predictors of serum free carnitine concentration in PD patients

Variables	Estimate	SE	*t*	95%CI		*p* Value
Age	−3.60	1.68	−2.13	−7.06	−0.13	0.041 *
Female	−0.94	1.45	−0.65	−3.91	2.02	0.520
Duration of dialysis	−0.15	0.06	−2.53	−0.28	−0.03	0.016 *
Body mass index	−0.02	0.18	−0.19	−0.24	0.19	0.849
Serum urea nitrogen	0.05	0.12	0.37	−0.21	0.31	0.716
Creatinine	0.60	0.71	0.85	−0.82	2.08	0.398
Hemoglobin	−0.78	1.50	−0.55	−3.82	2.25	0.603
Albumin	1.85	3.12	0.6	−4.45	8.12	0.557
Calcium	−0.94	1.72	−0.55	−4.41	2.53	0.587
Phosphate	−0.93	1.90	−0.49	−4.47	2.9	0.625
C-reactive protein	−0.67	1.42	−0.47	−3.55	2.22	0.472
HDL-cholesterol	−0.06	0.09	−0.73	−0.25	0.11	0.468
β_2_-MG	−0.28	0.18	−1.57	−0.65	0.08	0.123
Kt/V total	0.94	0.71	0.38	−4.04	5.92	0.703

β_2_-MG, β_2_-microglobulin; CI, confidence interval; HDL, high-density lipoprotein; SE, standard error. * *p* < 0.05.
